# Pulsed-field ablation of atrial fibrillation with a pentaspline catheter across National Health Service England centres

**DOI:** 10.1136/openhrt-2024-003094

**Published:** 2024-12-18

**Authors:** Mark T Mills, Saket Trivedi, Matthew J Lovell, Francis Murgatroyd, Peter Calvert, Vishal Luther, Dhiraj Gupta, Claire Martin, Sarah Zeriouh, Greg Mellor, Richard Balasubramaniam, Mark Sopher, Julian Boullin, Aruna Arujuna, Shajil Chalil, Scott Gall, Zhong Chen, Magdi Saba, Una Buckley, Riyaz Somani, Shui Hao Chin, David Jones, Riyaz A Kaba, Mark O'Neill, Tom Wong, Derick M Todd

**Affiliations:** 1 Liverpool Centre for Cardiovascular Science at University of Liverpool, Liverpool John Moores University and Liverpool Heart and Chest Hospital, Liverpool, UK; 2 Liverpool Heart and Chest Hospital NHS Foundation Trust, Liverpool, UK; 3 NHS England Cardiac Rhythm Management Device Working Group, London, UK; 4 Royal Devon University Healthcare NHS Foundation Trust, Exeter, UK; 5 King's College Hospital NHS Foundation Trust, London, UK; 6 Royal Papworth Hospital NHS Foundation Trust, Cambridge, UK; 7 University Hospitals Dorset NHS Foundation Trust, Bournemouth, UK; 8 Lancashire Cardiac Centre, Victoria Hospital, Blackpool Teaching Hospitals NHS Trust Foundation, Blackpool, UK; 9 Biomedical Engineering and Imaging Sciences Department, King’s College London, London, UK; 10 Royal Brompton and Harefield Hospitals, London, UK; 11 St George's University Hospitals NHS Foundation Trust, London, UK; 12 Guy's and St Thomas' NHS Foundation Trust, London, UK; 13 St Vincent’s Private Hospital, Dublin, Ireland; 14 University Hospitals of Leicester NHS Trust, Leicester, UK

**Keywords:** Atrial Fibrillation, Catheter Ablation, Electrophysiology, Ablation Techniques

## Abstract

**Introduction:**

Pulsed-field ablation (PFA) is a novel modality for pulmonary vein isolation in patients with atrial fibrillation (AF). We describe the initial uptake and experience of PFA using a pentaspline catheter across selected National Health Service England (NHSE) centres.

**Methods:**

Data collected by NHSE Specialised Services Development Programme regarding AF ablation procedures using a single-shot, pentaspline, multielectrode PFA catheter (FARAWAVE, Boston Scientific) between June 2022 and August 2024 were aggregated and analysed to examine procedural metrics, acute efficacy and safety outcomes over 3-month follow-up.

**Results:**

1034 procedures were submitted. The patients were 32.1% female, mean age 63.8±10.7 years, 53.1% paroxysmal AF and 89.7% first-time AF ablation. Procedures were performed by 48 consultant operators at nine NHSE centres, with a mean of 115 procedures per centre (range 25–264). 93.7% of procedures were performed under general anaesthesia. Median skin-to-skin procedure time was 74 min (IQR 55–96 min) and fluoroscopy time 20 min (IQR 15–27 min). Electroanatomical mapping was used in 15.3%. In first-time ablation cases, acute isolation of all pulmonary veins was achieved in 99.5% of patients. Left atrial (LA) posterior wall ablation using the PFA catheter was performed in 11.0% of cases; additional LA radiofrequency ablation was performed in 0.6%. The major and minor acute procedural complication rates were, respectively, 1.3% and 3.1%, with no reports of periprocedural death or atrio-oesophageal fistula. 63.8% of patients were discharged on the day of procedure. Follow-up data were available for 870 procedures (84.1%). In the 3 months following ablation, hospitalisation for arrhythmia occurred in 3.2%, with 0.9% rehospitalised for procedural-related complications.

**Conclusion:**

In this real-world, nationwide registry of a pentaspline PFA catheter, efficacy, safety and efficiency outcomes were comparable to those from previous PFA studies in patients with AF.

**WHAT IS ALREADY KNOWN ON THIS TOPIC**

- Pulsed-field ablation (PFA) is an emerging modality for performing catheter ablation of atrial fibrillation (AF).

**WHAT THIS STUDY ADDS**

- In this mandatory, nationwide report of AF ablation procedures using a single-shot, pentaspline PFA ablation catheter, clinical outcomes were comparable to those from early clinical studies, highlighting its safety and efficacy in a real-world cohort.

**HOW THIS STUDY MIGHT AFFECT RESEARCH, PRACTICE OR POLICY**

- These data support the clinical use of the single-shot, pentaspline PFA catheter, although detailed cost-effectiveness analyses comparing it to established technologies are required.

## Introduction

 In patients with atrial fibrillation (AF), pulsed-field ablation (PFA) is an emerging ablation modality for achieving pulmonary vein isolation (PVI). Rather than applying thermal energy, as is the case with radiofrequency (RF) and cryoablation, PFA applies an electrical field, leading to selective myocardial cellular injury through electroporation.^1^ Early clinical studies suggest that PFA performs similarly to thermal ablation in terms of acute-term and medium-term efficacy, with purported safety and efficiency advantages.^2^

The FARAPULSE system (Boston Scientific), comprising a single-shot, pentaspline, multielectrode catheter (FARAWAVE) and a pulsed-field energy generator, was the first PFA system to become commercially available in Europe. In England, FARAPULSE obtained regulatory approval in mid-2022, but reimbursement within the National Health Service (NHS) in England was limited to centres participating in an evaluation scheme, which involved mandatory data collection for all procedures.

While single-centre data of PFA in the NHS are starting to emerge,^3^ national data are lacking. In the present study, we analysed the mandated data to describe the initial uptake and experience of FARAPULSE AF ablation across NHS England (NHSE) centres since its introduction in 2022. Specifically, we aimed to determine whether real-world, acute outcomes of FARAPULSE within a national, publicly funded healthcare system were comparable to those from international studies.^2 4^

## Methods

### Study design

For business and commissioning purposes, an observational registry of FARAPULSE AF ablation procedures was created by the Cardiac Rhythm Management Device Working Group (DWG) on behalf of the NHSE Specialised Services Development Programme.

During the evaluation period (starting in June 2022), FARAPULSE was only made available to participating centres. Potential centres were proposed by the manufacturer and approved as suitable by the DWG. Centres were required to submit data for all consecutive cases, and reimbursement of device costs was dependent on compliance. The purposes of the registry were to evaluate the safety and acute efficacy of this new technology and to collect some information regarding resource utilisation. The participating centres were Blackpool Teaching Hospitals, Glenfield Hospital, Harefield Hospital, Liverpool Heart and Chest Hospital, Royal Bournemouth Hospital, Royal Brompton Hospital, Royal Papworth Hospital, St George’s Hospital and St Thomas’ Hospital. A phased rollout occurred across centres, with the first centre starting in June 2022 and the ninth centre in October 2023. Centres returned their completed datasets between January and August 2024. As such, while the data collection captured consecutive cases from the introduction of FARAPULSE in each centre, the date of last case submission varied between centres.

Each centre’s dataset was maintained by NHSE, with patient-identifiable data pseudonymised (to preserve anonymity while permitting each centre to check and amend its data). The consolidated data were only available to individuals directly employed by the Specialised Services Devices Programme and a limited number of clinician members of the DWG (MTM, ST, MJL, FM and DT). These individuals, who take responsibility for the integrity of the consolidated dataset, performed all data cleaning and analysis, to produce an initial report for NHSE internal commissioning purposes, and a subsequent further analysis for the purpose of this article (with permission from NHSE and lead clinicians at each centre). Only aggregated summary data were provided to the wider authorship group.

Patient and public involvement was not sought in the design or writing of this manuscript.

### Ablation procedures

Routine informed, written consent for ablation procedures was obtained from patients. Both first-time and redo AF ablation procedures were included. PFA was performed using the FARAWAVE catheter as per manufacturer recommendations, with a minimum of eight applications per PV (four in the ‘basket’ and four in the ‘flower’ configuration). Data for adjunctive non-PV PFA for the treatment of AF (eg, posterior wall isolation (PWI)), as well as procedures involving additional thermal ablation (eg, additional RF cavotricuspid isthmus ablation (CTI)), and those in whom electroanatomical mapping was used, were also included. Data on FARAWAVE catheter size (ie, 31 or 35 mm) were not collected. Anaesthetic method, transoesophageal echocardiography, periprocedural anticoagulation and same-day discharge protocols were at the discretion of the participating centres and operators.

### Outcome measures

Our approach examined five areas: (1) centre and operator case numbers, (2) baseline patient demographics, (3) procedural metrics and efficacy outcomes; (4) acute procedural safety outcomes and (5) short-term follow-up outcomes. The data fields recorded were selected by the NHSE Specialised Services Devices Programme in line with the Caldicott principles, whereby only the minimum necessary confidential and non-patient-identifiable data were collected.^5^

Baseline demographics included age, sex, AF type, prior AF ablation and the presence of associated structural heart disease. Procedural metrics and efficacy outcomes included equipment use, catheter lab occupancy time, skin-to-skin procedure time, fluoroscopy time, periprocedural anticoagulation strategy, anaesthetic method, use of transoesophageal echocardiography and electroanatomical mapping, acute procedural success (defined as entrance and/or exit block of all identified PVs in first-time AF ablation cases), additional non-PV ablation, the use of additional thermal ablation and length of hospital stay. Acute safety outcomes were classified into six categories (pericardial, stroke, coronary, arrhythmic, vascular and other) and divided into major and minor complications. Complications that were life-threatening, requiring intervention or resulting in permanent disability or death were classed as major. Short-term follow-up included hospitalisation within 3 months for atrial arrhythmia or procedure-related complications (to allow for late-presenting complications such as atrio-oesophageal fistula). The long-term efficacy of the procedure, either in terms of arrhythmia recurrence or symptoms, was beyond the scope of this registry.

### Statistical analysis

Statistical distribution was determined with visual histogram inspection and the Shapiro-Wilk test. Categorical variables were expressed as counts and percentages. Depending on the distribution, continuous variables were expressed as mean±SD or median (IQR). For comparisons between two groups, continuous variables were compared using t-tests or non-parametric equivalents. Two-sided p values <0.05 were considered statistically significant. Statistical analysis was performed in SPSS (version 29.0.1.0) and R.

## Results

### Case numbers

**Figure 1:**
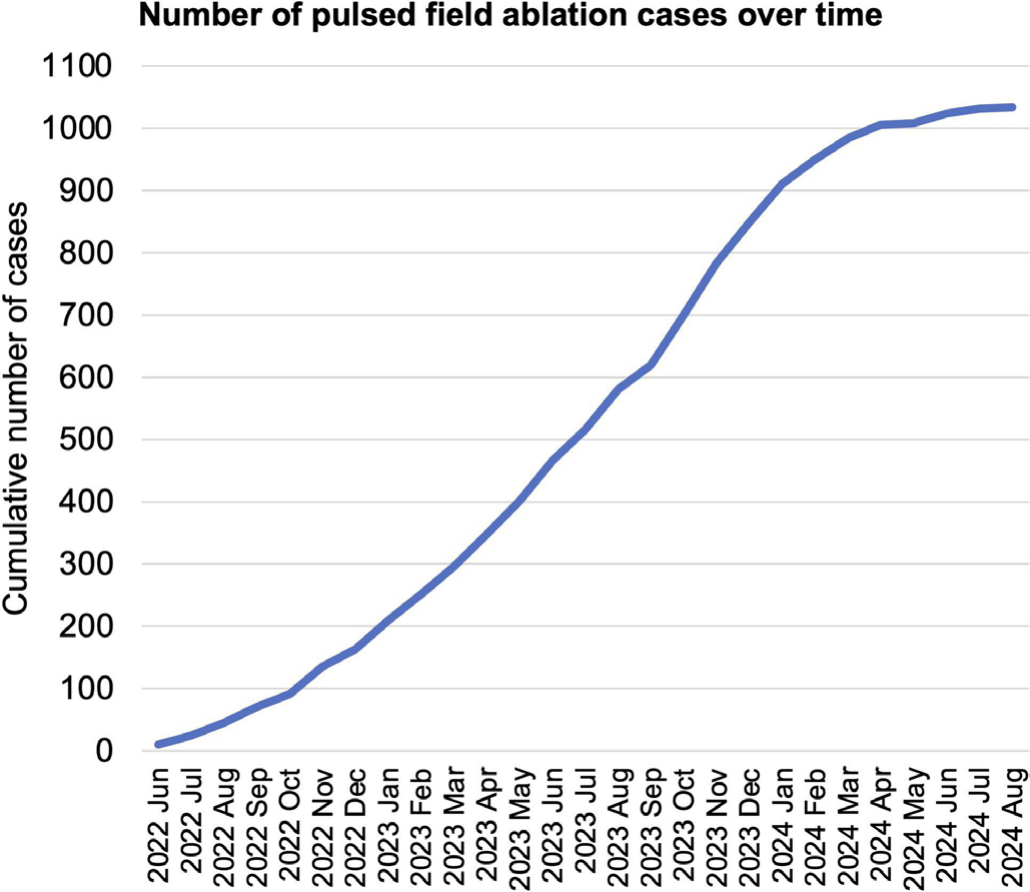
Cumulative atrial fibrillation pulsed-field ablation case numbers over time across all centres included in the report. Notably, centres returned their completed data between January and August 2024. As such, while the data collection captured consecutive cases from the introduction of FARAPULSE in each centre, the date of last case submission varied between centres. This is reflected in the flattening of the curve from January 2024 onwards.

Nine centres reported 1034 FARAPULSE AF ablation procedures during the submission period, with a mean of 115 procedures per centre (range 25–264). Cumulative case numbers over time are presented in figure 1. A total of 48 consultant operators performed the procedures, with a median of 19 procedures per operator (IQR 9–31) (table 1).

**Table 1:** Number of pulsed-field ablation (PFA) operators per centre and average number of PFA procedures per operator

Centre	Number of PFA procedures(n)	Number of consultant operators(n)	Number of PFA procedures per operator(median (IQR))
A	264	9	22 (21–34)
B	183	5	46 (18–50)
C	176	6	31 (23–39)
D	103	5	17 (14–27)
E	103	6	17 (5–29)
F	71	4*	17 (13–22)
G	56	4	10 (6–18)
H	53	7	6 (4–11)
I	25	3*	9 (8–9)
All centres	1034	48[Table-fn T1_FN1]	19 (9–31)

* One consultant operator performed procedures at two hospitals (included towards the count for both centres, but included only once for the total number of consultant operators).

### Baseline demographics

Patient demographics are summarised in table 2. Across the 1034 cases, mean age was 63.8±10.7 years, and 32.1% of patients were female. Notably, female patients were older than male patients (66.0±9.7 years vs 62.8±11.0, p<0.001). AF type was paroxysmal in 53.1% (n=549), persistent in 46.7% (n=483) and not documented in two cases. 89.7% of procedures (n=927) were for first-time AF ablation. 13.6% (n=141) had a left ventricular ejection fraction <45%, 4.4% (n=45) had significant mitral valve disease, and 1.9% (n=20) had significant aortic valve disease. 98.2% (n=1015) of patients followed an uninterrupted anticoagulation strategy (96.1% on direct oral anticoagulants and 2.0% on vitamin K antagonists).

**Table 2:** Baseline patient demographics

	Patients undergoing PFA(n=1034)
Age, mean±SD (years)	63.8±10.7
Female, n (%)	332 (32.1)
AF type, n (%)	
Paroxysmal	549 (53.1)
Persistent	483 (46.7)
Not stated	2 (0.2)
First-time AF ablation, n (%)	927 (89.7)
LVEF<45%, n (%)	141 (13.6)
Hypertrophic cardiomyopathy, n (%)	10 (1.0)
Mitral valve disease	45 (4.4)
Aortic valve disease	20 (1.9)
OAC prior to ablation, n (%)	1015 (98.2)
DOAC	994 (96.1)
Warfarin	21 (2.0)
LAAO device in situ, n (%)	2 (0.2)
ASD closure device in situ, n (%)	3 (0.3)

AFatrial fibrillationASDatrial septal defectDOACdirect oral anticoagulantLAAOleft atrial appendage occlusionLVEFleft ventricular ejection fractionPFApulsed-field ablation

### Procedural metrics and efficacy outcomes

Procedural metrics and acute efficacy outcomes are summarised in table 3 for the entire cohort and individual centres.

**Table 3:** Procedural metrics and efficacy outcomes of pulsed-field ablation of atrial fibrillation in NHS England centres

Centre	Number of procedures(n)	General anaesthesia(n, (%))	Lab occupancy time(min)	Skin-to-skin procedure time(min)	Fluoroscopy time(min)	Electro-anatomical mapping (n, (%))	Acute success in first-time PVI cases(%)	Same-day discharge(n, (%))
A	264	253 (95.8)	104 (91–123)	72 (60–89)	19 (15–25)	0 (0)	260/260 (100%)	133 (50.4)
B	183	183 (100)	115 (96–159)	95 (77–117)	21 (18–29)	38 (20.8)	145/145 (100%)	165 (90.2)
C	176	176 (100)	114 (95–130)	75 (57–95)	22 (17–29)	15 (8.5)	157/160 (98.1%)	164 (93.2)
D	103	103 (100)	115 (86–145)	80 (53–110)	22 (14–30)	24 (23.3)	78/80 (97.5)	45 (43.7)
E	103	49 (47.6)	112 (92–138)	74 (56–100)	21 (17–31)	23 (22.3)	95/95 (100)	51 (49.5)
F	71	71 (100)	93 (76–108)	53 (45–67)	15 (12–22)	1 (1.4)	71/71 (100)	42 (59.2)
G	56	56 (100)	120 (103–150)	101 (81–124)	20 (16–25)	54 (96.4)	36/36 (100)	3 (5.4)
H	53	53 (100)	83 (65–101)	40 (30–54)	14 (10–19)	1 (1.9)	52/52 (100)	46 (86.8)
I	25	25 (100)	132 (110–145)	68 (55–85)	17 (16–21)	2 (8.0)	24/24 (100)	11 (44.0)
All centres	1034	969 (93.7)	109 (90–135)	74 (55–96)	20 (15–27)	158 (15.3)	918/923 (99.5)	660 (63.8)

NHSNational Health ServicePVIpulmonary vein isolation

In the majority of cases (72.1%, n=745), a single diagnostic electrophysiology catheter was used. 41.0% (n=424) of patients were in atrial arrhythmia at procedure start. The majority (93.7%, n=969) of cases were performed under general anaesthesia, with 5.3% (n=55) performed under propofol deep sedation without endotracheal intubation, and 1.0% (n=10) under conscious sedation using opiates and benzodiazepines. Transoesophageal echocardiography was undertaken in 49.8% (n=515) of cases, and electroanatomical mapping used in 15.3% (n=158). Mapping systems used were RHYTHMIA (Boston Scientific; n=73), EnSite/Nav X (Abbott; n=40), Carto (Biosense Webster; n=28) and not specified in 17 patients.

Across all cases, median lab occupancy time was 109 min (IQR 90–135 min), skin-to-skin time was 74 min (55–96 min) and fluoroscopy time was 20 min (15–27 min). Skin-to-skin and fluoroscopy times were longer in patients who underwent electroanatomical mapping than in those who did not (skin-to-skin procedural time, respectively, 105 min (85–138 min) vs 67 min (52–89 min), p<0.001; fluoroscopy time: 25 min (19–35 min) vs 19 min (14–25 min), p<0.001).

**Figure 2:**
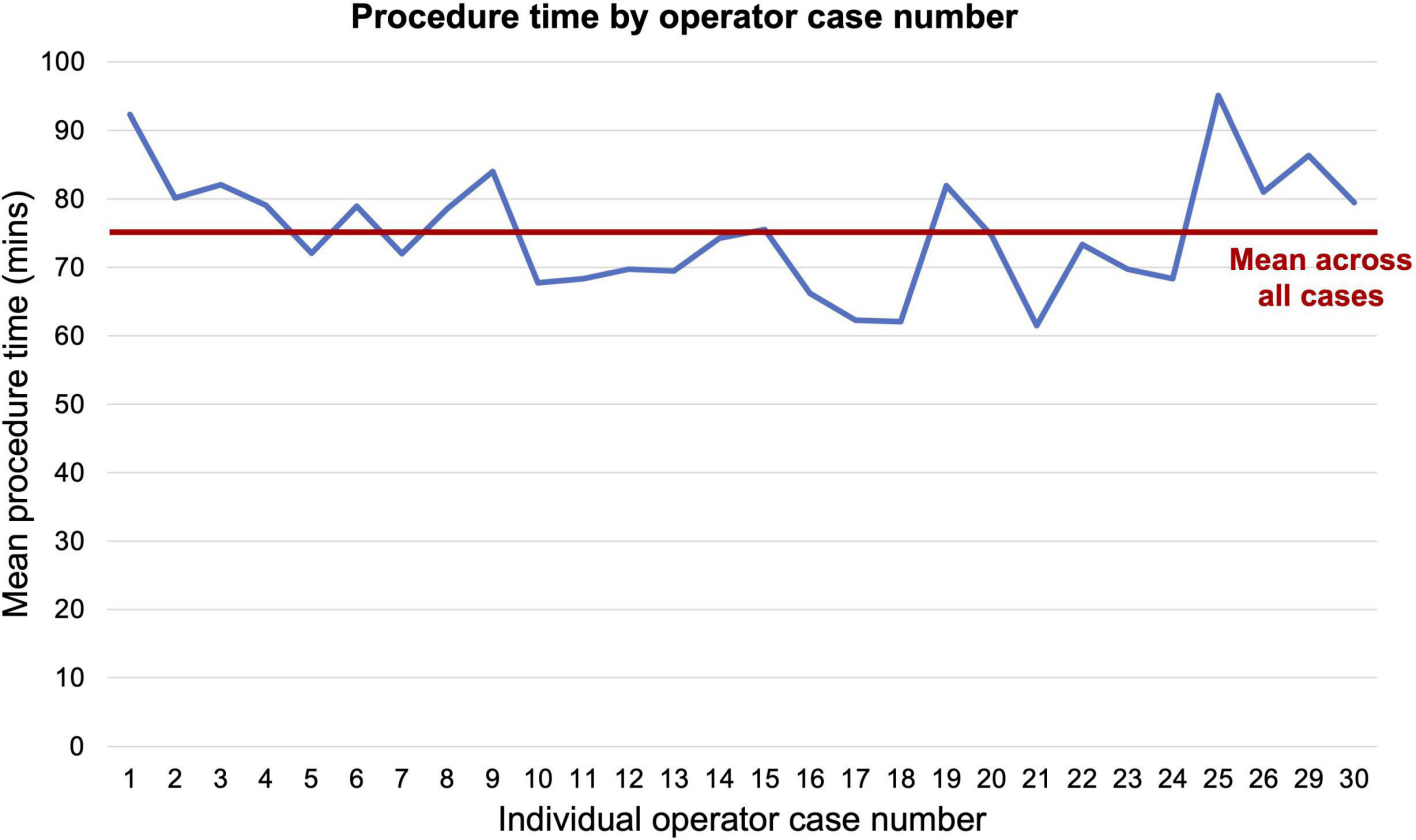
Mean skin-to-skin procedure time by operator case number. Data presented for cases of pulmonary vein isolation alone in the 25 consultant operators who performed at least 15 procedures. Case 1=operator’s first FARAPULSE PVI case; Case 30=operator’s 30th case. For operators performing more than 30 cases, data censored at the operator’s 30th case to ensure a minimum of 10 operators per case number. PVI, pulmonary vein isolation.

25 consultant operators performed at least 15 procedures. Across these operators, mean procedure time by operator case number is shown in figure 2, indicating that the procedure can be taken up by experienced operators without a significant learning curve.

PVI alone was performed in 82.1% (n=849) of cases. Left atrial (LA) PWI using the FARAWAVE catheter was performed in 11.0% (n=114) of cases, while additional LA RF ablation was performed in 0.58% (n=6) of cases. The latter comprised four cases of PWI, one case of PWI with mitral line and complex fractionated atrial electrogram ablation and one case of ostial right upper PV touch-up following PFA. RF CTI ablation was performed in 6.0% of cases (n=62), RF slow pathway ablation in 0.2% (n=2) and unspecified right atrial RF ablation in 0.6% (n=6) cases. FARAWAVE CTI ablation was attempted in one case, resulting in transient 2:1 atrioventricular block without successful CTI.

Rate of acute PVI success was reported in 923/926 patients. In these, isolation of all identified PVs was achieved in 99.5% of cases (918/923), with 99.8% of all identified PVs (3689 out of 3695) isolated.

Same-day discharge occurred in 63.8% (n=660) of cases, with 33.1% (n=343) discharged the day after their procedure. Marked variations in same-day discharge rates were noted across centres, ranging from 5.4% to 93.2% (table 3).

### Acute procedural safety outcomes

The overall acute procedural complication rate was 4.4% (45 complications across 1034 cases). Of these, 1.3% (n=13) were major and 3.1% (n=32) minor. Complications are summarised in table 4.

**Table 4:** Acute procedural complications following pulsed-field ablation of atrial fibrillation across nine NHS England centres

Acute complications	N (%)Total cohort, n=1034
Pericardial	7 (0.68)
Major	3 (0.29)
Minor	4 (0.39)
Cerebrovascular	3 (0.29)
Major	3 (0.29)
Minor	0
Coronary	1 (0.10)
Major	1 (0.10)
Minor	0
Arrhythmic	9 (0.87)
Major	3 (0.29)
Minor	6 (0.58)
Vascular	16 (1.55)
Major	2 (0.19)
Minor	14 (1.35)
Other	9 (0.87)
Major	1 (0.10)
Minor	8 (0.77)
Mortality	0
Total	45 (4.35)
Major	13 (1.26)
Minor	32 (3.09)

NHSNational Health Service

The major complications included cardiac tamponade requiring pericardiocentesis (n=3), stroke (n=2), transient ischaemic attack (n=1), acute coronary syndrome requiring intervention (n=1), sinus or atrioventricular node disease with bradycardia requiring temporary (n=1) or permanent (n=2) pacemaker implantation, vascular injury requiring intervention (n=2) and embolisation of a LA appendage occlusion (LAAO) device (in a patient undergoing concomitant PFA and LAAO insertion, n=1).

Minor complications included inadvertent pericardial access or the development of pericardial effusion without the need for pericardiocentesis (n=4), transient bradycardia not requiring intervention (n=6), vascular injury delaying discharge but not requiring intervention (n=14), periprocedural hypotensive episodes (n=7) and haemoptysis of undetermined cause (n=1).

No cases of periprocedural mortality, phrenic nerve palsy or oesophageal injury were reported.

### Short-term follow-up outcomes

Follow-up data were available for 870 (84.1%) patients. In the first 3 months following ablation, hospitalisation for atrial arrhythmia occurred in 3.2% (28/870) of patients, with a median length of hospital stay of 1 night (1–2 nights). Hospitalisation for procedure-related complication(s) occurred in 0.9% (8/870), with a median length of stay of 2 nights (1–3 nights). Complications during follow-up included stroke (n=2), transient ischaemic attack (n=1), acute coronary syndrome requiring intervention (n=1), sinus node disease requiring permanent pacemaker implantation (n=1), vascular injury managed conservatively (n=2) or requiring intervention (n=2). Four patients (0.4%) died within 3 months of ablation, although none of the deaths were deemed to be related to the ablation or procedural-related complications. No cases of atrio-oesophageal fistula were reported during follow-up.

## Discussion

Our study reports real-world outcomes of AF ablation using a pentaspline PFA catheter in an unselected cohort of patients in England since its introduction in 2022. The main findings of our analysis are (1) over the study period, 1034 procedures were performed across nine centres; (2) median skin-to-skin and fluoroscopy times, were, respectively, 74 and 20 min; (3) in first-time ablation cases, PVI was achieved in 99.5% of cases; (4) LA posterior wall ablation using the PFA catheter was performed in 11.0% of cases; (5) The major and minor acute procedural complication rates were, respectively, 1.3% and 3.1% and (6) in the first 3 months following ablation, hospitalisation for arrhythmia occurred in 3.2%, with 0.9% rehospitalised for procedural-related complications.

### Comparison with published studies

While regulatory approval studies are crucial for the assessment and introduction of novel AF ablation technologies, postmarket release analyses are key to identifying real-world outcomes, particularly with regard to acute procedural efficacy, safety and efficiency. In our study, these metrics were comparable to those from landmark FARAPULSE trials and registries,^2 4 6^ highlighting the generalisability of this modality to a wider clinical setting.

In the ADVENT randomised controlled trial, 607 patients were randomised 1:1 to PFA or thermal ablation, with an acute PFA PVI success rate of 99.6%, mean skin-to-skin procedure time of 106±29 min, fluoroscopy time of 21±11 min and major complication rate of 2.0%.^2^ Similarly, the EU-PORIA registry retrospectively assessed 1233 patients receiving PFA across seven European centres, with an acute PVI success rate of 99.96%, median skin-to-skin procedure time of 58 (40–87) min, fluoroscopy time of 14 (9–21) min and 1.7% major complication rate.^4^ Most recently, the MANIFEST-17k registry reported safety outcomes in 17 642 patients undergoing PFA across 20 countries, with major and minor complication rates of 0.98% and 3.21%, respectively.^4^ Overall, our findings are similar to those from ADVENT, EU-PORIA and MANIFEST-17k, and notably, demonstrated high consistency across centres (table 3). Further, the 0.9% 3-month hospitalisation rate for procedural-related complications was comparable to published thermal ablation studies,^7 8^ with low rates of hospitalisation for arrhythmia compared with the published literature.^9^

Rates of PWI using the pentaspline PFA catheter in our study (11%) were similar to those in other registries, such as EU-PORIA.^4^ Indeed, given the purported safety of PFA in proximity to the oesophagus, PWI has seen a recent uptake in centres internationally.^10 11^ We must highlight, however, that this approach is outside of the current labelled indications for the FARAPULSE system, and that guidelines recommend reserving extra-PV ablation to select patients only,^12^ especially given reports of haemolysis with acute renal failure if excessive PFA is delivered.^12^

An important difference in our registry was the low use of electroanatomical mapping (15.3%, compared with 92.5% in ADVENT,^2^ 29% in MANIFEST-PF^6^ and 33% in EU-PORIA).^4^ To date, mapping has not been shown to reduce AF recurrence following PFA.^13^ Although integration of the FARAPULSE system into electroanatomical mapping systems has been shown to reduce fluoroscopy exposure in some studies,^14^ mapping was associated with increased fluoroscopy times in our registry. This may be explained by the learning curve associated with mapping during FARAPULSE cases, and its use in guiding non-PV ablation. Future studies examining the impact of mapping on long-term efficacy and cost-effectiveness are required.

### Potential advantages of PFA in the NHS

As the incidence of AF rises in England^15^ and internationally,^16^ adherence to a holistic treatment pathway—focused on stroke risk reduction, symptom control and comorbidity management—is critical to improving clinical outcomes.^17^ AF ablation is an evidence-based, guideline-recommended treatment strategy in patients with drug-refractory symptoms or AF-related left ventricular dysfunction.^12^ According to recent estimates from the National Cardiac Audit Programme, around 11 000 AF ablation procedures are performed in England per year, having increased from 8000 over the last decade.^18^ Novel technologies that have the potential to improve safety, efficacy, and/or efficiency and cost-effectiveness of AF ablation, therefore, warrant careful consideration.

From a safety perspective, the cardioselectivity of PFA is frequently cited as its main advantage over thermal ablation.^19^ However, claims of cardioselectivity should be embraced with vigilance, as transient phrenic nerve palsy has been described in large registries (eg, in 11 of 17 642 patients in the MANIFEST-17k registry^4^) and case reports.^20 21^ Although rare, the incidence of atrio-oesophageal fistula is thought to be around 1 in 2600 with RF ablation and 1 in 66 000 with cryoablation.^21^ To date, no such cases have been reported with PFA; given the increasing use of PFA on the LA posterior wall, these results are encouraging, although not definitive. Coronary spasm is a recognised phenomenon during PFA.^4^ In our registry, one patient (0.1%) suffered a myocardial infarction periprocedurally, requiring intervention. Established recognised complications of AF ablation including cardiac tamponade (0.3%), stroke (0.2%) and transient ischaemic attack (0.1%) continue to occur with this technology.

Alongside safety, efficacy—both in terms of acute and long-term outcomes—is paramount. Although our study did not assess for long-term AF recurrence, acute procedural success was high and consistent across centres. These high success rates, which were noted from the first use of the pentaspline catheter across operators and centres, suggest that the learning curve is rapid in consultant operators with experience in thermal AF ablation. This offers the promise of a short, safe and effective procedure across operators, in comparison with the steeper learning curve often associated with RF ablation. The shared skillset of FARAPULSE PVI with cryoablation might explain the absence of a learning curve, although data on consultant operator experience of other ‘single-shot’ devices were not available for analysis. Of note, randomised studies have shown no differences in long-term outcomes between PFA and thermal ablation,^2 3^ suggesting that this metric alone should not encourage the use of one modality over another.

Another area where PFA may prove advantageous over thermal ablation is procedural efficiency. Indeed, in the ADVENT trial, PFA was associated with significantly shorter procedure times than thermal ablation (105 vs 123 min; including mapping in the majority of cases). In our study, median lab occupancy time was 109 min suggesting that, in a standard 8-hour working day, an average of four FARAPULSE procedures can be performed. In centres where three AF ablation cases are currently listed per day, this could increase throughput, without compromising safety or efficacy.

### Potential limitations of PFA in the NHS

Two important factors may limit the widespread adoption of PFA in the NHS. First, PFA is usually performed under general anaesthesia or deep sedation with propofol (respectively, 93.7% and 5.3% in our study). In England, as in the majority of countries worldwide, these approaches mandate anaesthetic support.^22^ Unfortunately, across many UK centres, general anaesthesia availability is limited and this may limit the more widespread adoption of PFA within the NHS.

Second, in a recent UK study, PFA procedures were found to result in higher overall procedure costs than RF or cryoablation (respectively, £10 010 vs £8949 vs £8106).^3^ In a publicly funded healthcare system such as the NHS, differences in costs are important, particularly when long-term effectiveness appears similar. Nevertheless, formal cost-effectiveness studies, taking into account a range of factors (such as AF recurrence, repeat hospitalisation, repeat procedures and patient-reported outcomes) have not yet been performed.

### Limitations

We acknowledge the limitations of our study, primarily that this was an observational analysis without a control group. As such, our data should be used only to ascertain descriptive procedural metrics and outcomes in a real-world cohort of patients undergoing FARAPULSE ablation in the NHS and not to draw comparisons between FARAPULSE and other PFA or thermal ablation systems. Data were limited to that collected by the Specialised Services Devices Programme, with certain demographics (eg, body mass index, antiarrhythmic drug therapy), procedural characteristics (eg, ultrasound-guided access, the number of PFA applications on the posterior wall) and follow-up outcomes (eg, recurrence of atrial arrhythmia not requiring hospitalisation) not reported. Despite strict guidance on data input, inconsistencies in individual centre reporting may have occurred; to mitigate this, centres were contacted during the data cleaning and analysis phase if statistical outliers were noted or clarifications were required.

## Conclusion

The use of the FARAPULSE system to perform AF ablation in England has gained momentum since 2022, with 1034 cases captured at nine centres in this study. In this real-world, nationwide registry, efficacy, safety and efficiency outcomes were comparable to those from published international studies. Potential advantages of FARAPULSE AF ablation in the NHS include a consistent efficacy and safety among operators, with no evidence of a learning curve and favourable procedural efficiency. However, widespread adoption may be limited by lack of general anaesthesia availability and cost. Detailed cost-effectiveness analyses comparing FARAPULSE to established thermal ablation technologies and emerging PFA systems are required.

## Data Availability

No data are available.
